# Factors Affecting Intention to Receive and Self-Reported Receipt of 2009 Pandemic (H1N1) Vaccine in Hong Kong: A Longitudinal Study

**DOI:** 10.1371/journal.pone.0017713

**Published:** 2011-03-11

**Authors:** Qiuyan Liao, Benjamin J. Cowling, Wendy Wing Tak Lam, Richard Fielding

**Affiliations:** 1 Health Behaviours Research Group, Department of Community Medicine and School of Public Health, The University of Hong Kong, Hong Kong Special Administrative Region, China; 2 Infectious Diseases Research Group, Department of Community Medicine and School of Public Health, The University of Hong Kong, Hong Kong Special Administrative Region, China; University of Liverpool, United Kingdom

## Abstract

**Background:**

Vaccination was a core component for mitigating the 2009 influenza pandemic (pH1N1). However, a vaccination program's efficacy largely depends on population compliance. We examined general population decision-making for pH1N1 vaccination using a modified Theory of Planned Behaviour (TBP).

**Methodology:**

We conducted a longitudinal study, collecting data before and after the introduction of pH1N1 vaccine in Hong Kong. Structural equation modeling (SEM) tested if a modified TPB had explanatory utility for vaccine uptake among adults.

**Principal Findings:**

Among 896 subjects who completed both the baseline and the follow-up surveys, 7% (67/896) reported being “likely/very likely/certain” to be vaccinated (intent) but two months later only 0.8% (7/896) reported having received pH1N1 vaccination. Perception of low risk from pH1N1 (60%) and concerns regarding adverse effects of the vaccine (37%) were primary justifications for avoiding pH1N1 vaccination. Greater perceived vaccine benefits (β = 0.15), less concerns regarding vaccine side-effects (β = −0.20), greater adherence to social norms of vaccination (β = 0.39), anticipated higher regret if not vaccinated (β = 0.47), perceived higher self-efficacy for vaccination (β = 0.12) and history of seasonal influenza vaccination (β = 0.12) were associated with higher intention to receive the pH1N1 vaccine, which in turn predicted self-reported vaccination uptake (β = 0.30). Social norm (β = 0.70), anticipated regret (β = 0.19) and vaccination intention (β = 0.31) were positively associated with, and accounted for 70% of variance in vaccination planning, which, in turn subsequently predicted self-reported vaccination uptake (β = 0.36) accounting for 36% of variance in reported vaccination behaviour.

**Conclusions/Significance:**

Perceived low risk from pH1N1 and perceived high risk from pH1N1 vaccine inhibited pH1N1 vaccine uptake. Both the TPB and the additional components contributed to intended vaccination uptake but social norms and anticipated regret predominantly associated with vaccination intention and planning. Vaccination planning is a more significant proximal determinant of uptake of pH1N1 vaccine than is intention. Intention alone is an unreliable predictor of future vaccine uptake.

## Introduction

Influenza contributes significantly to worldwide morbidity and mortality [1]. Periodically, influenza viruses mutate into antigenically-different strains leading to global pandemics [2]. The 2009 influenza pandemic (pH1N1) was caused by a triple reassortment of human, swine and avian influenza viruses [3]. Vaccination is the most effective intervention for preventing influenza [4] and a core part of national pandemic plans for pandemic mitigation. Lead times of at least 6 months in producing a vaccine against a novel strain means that while vaccines may be unavailable in time to prevent the first wave of a pandemic [5], [6], effective public uptake of a vaccine may mitigate subsequent waves [7].

### Background

**Figure 1:**
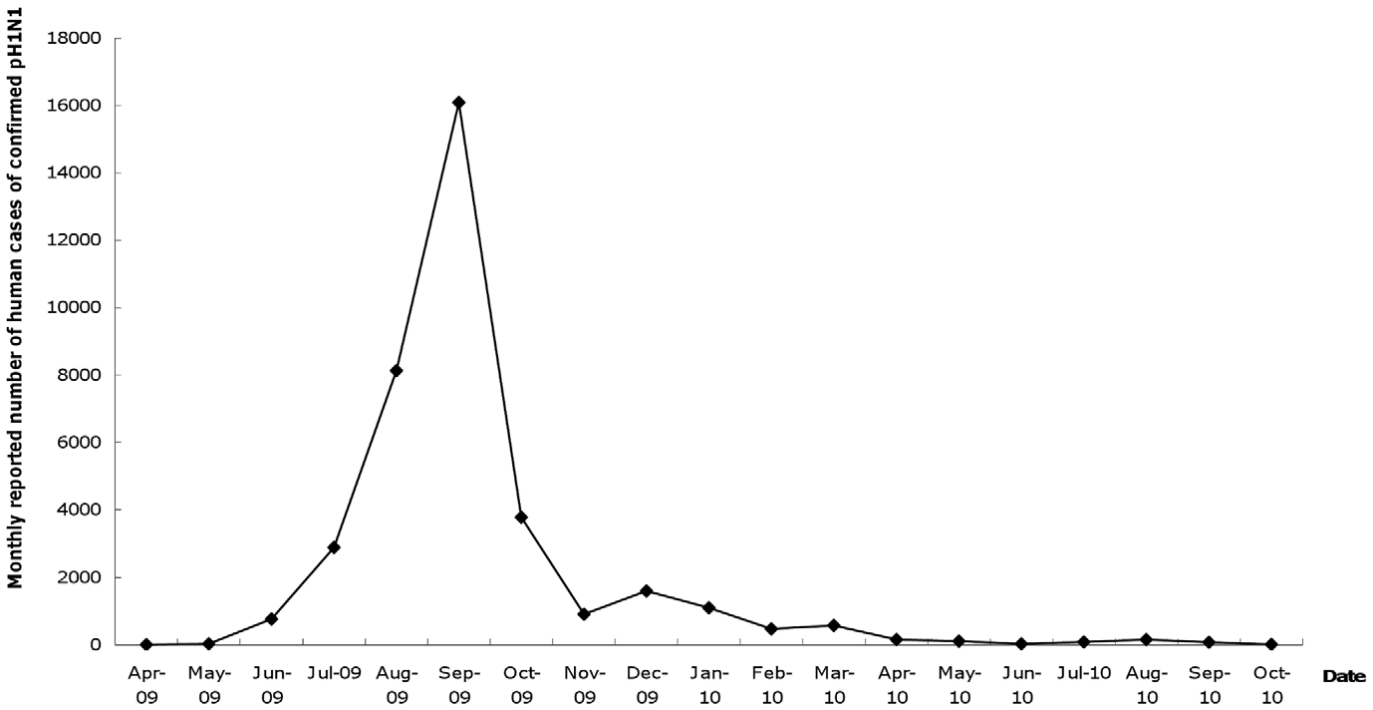
Epidemic curve showing the monthly reported human pH1N1 cases in Hong Kong. Data source: Center for Health Protection Hong Kong government. Available at http://www.chp.gov.hk/en/dns_submenu/10/26.html.

Significant health promotion activities regarding influenza prevention have been prominent in Hong Kong since well before the onset of pH1N1, arising largely from the Severe Acute Respiratory Infection (SARS) epidemic and A/H5N1 Bird Flu outbreaks. Seasonal influenza vaccination is widely promoted each year. Hong Kong's pH1N1 epidemic started on 11 June 2009, peaking in September, and by early November had petered out (Figure 1). By the end of December 2009, the Hong Kong government had recorded 37,174 human pH1N1 cases [8] in a population of ∼7 million. To minimize any potential second wave, significant televised and other publicity was given to the launch of a pH1N1 vaccination programme on 21 December 2009, initially for five priority groups: healthcare workers, persons with chronic illness and pregnant women, children aged 6 months to 6 years, adults aged 65 years or above, and pig farmers and slaughtering industry personnel [9]. On 26 January 2010 pH1N1 vaccination was extended to the general public. The vaccination was free for priority group members [10], but cost HK$100–150 (US$13–20, 1–1.5% of Hong Kong's median monthly income of HK$10,000/US$1,286/€991) per dose for the general population. A study in July 2009 of 301 respondents projected that vaccine uptake would be influenced by end-user cost, with 45%, of Hong Kong's general population being “highly likely” to take up pH1N1 vaccine if free, and 24% if costing HK$100–200 (US$13–25) [11], [12].

**Figure 2:**
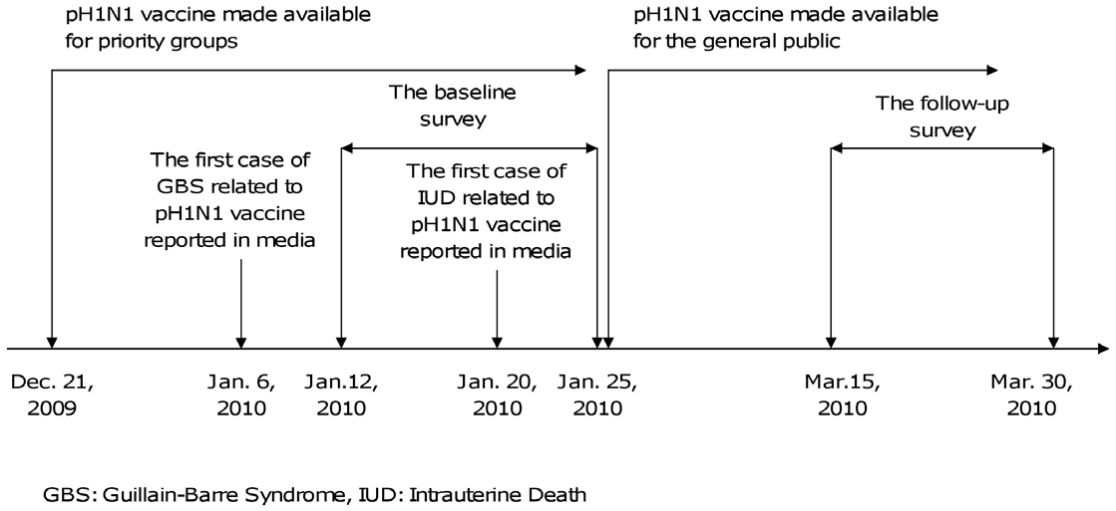
A chronology of events about the pH1N1vaccine availability, reporting of potential vaccine-related adverse events and conducting of the current study.

From November 2009 onwards, television, radio, newspaper and official websites strongly encouraged priority groups to have pH1N1 vaccination [13]. However, the Hong Kong government did not make recommendations for the general population, who were asked to judge for themselves whether to be vaccinated or not. Shortly after the vaccine launch for priority groups, local media prominently attributed several adverse events to pH1N1 vaccination, including, a case of Guillain-Barre Syndrome (GBS) diagnosed a week after pH1N1 vaccination, reported on 6th January 2010, and an intrauterine death (IUD) 3 weeks following the mother's vaccination, reported on 20th January 2010 (Figure 2). In both cases local health agencies presented convincing evidence challenging the link between vaccination and the two adverse events but were largely ignored. Retrospectively, a drop in pH1N1 vaccination uptake among priority groups was observed [14]. It seems probable that the adverse media reports had impeded vaccination uptake among general population. We collected baseline data between 12–25 January, 2010, immediately before pH1N1 vaccine was made available to the general population and then two months later (15–30 March 2010) we recorded their reported vaccination status (Figure 2) with the intention of modelling how general population decision-making regarding pH1N1 vaccination might predict subsequent vaccine uptake.

### Previous findings and knowledge gap

Empirical studies have found that history of seasonal influenza vaccination [12], [15]–[18], perceived risk of pandemic influenza [17], [19]–[26], worry [17], [22], [26], [27], and attitudes towards vaccine, such as vaccine efficacy and side-effects [12], [15], [20], [24]–[26] were significantly associated with intention to receive a vaccine against the influenza pandemic. This is consistent with the findings related to determinants of vaccination against seasonal influenza [28]–[32]. However, there are some common and significant limitations to these empirical studies. First, all except one [24] relied on vaccination intention to predict the actual vaccination uptake. In one study, since only a few respondents reported having received the pH1N1 vaccine, the authors combined those intending to get vaccinated with those who had already received the vaccine into one “intending” group and examined factors associated with this ‘vaccination intention’ [20]. This is problematic because factors associated with vaccination intention and actual vaccination receipt probably differ. Moreover, the reliability of intention as a predictor of actual behavior remains controversial. Harris et al. found that only about half of “intending” recipients of seasonal influenza vaccination actually take it and almost all those who do not intend to take it remained unvaccinated [33]. Moreover, most studies conducted before the pandemic occurred or before the vaccine was available [11], [12], [16]–[19], [21], [23] reported relative high intentions for vaccination against pandemic influenza among study respondents. For example, in April–May, 2009, 65.5% and 94.6% of Dutch respondents reported intending to take pH1N1 vaccination prior to or at the onset of the (potential) pandemic phase, respectively [23]. Similarly, in Hong Kong 45% of 301 respondents in July 2009 reported being “highly likely” to receive pH1N1 vaccine if offered for free [11], [12]. However, by the time vaccination became available intention appeared much lower with only 10–15% of study respondents in France and in Turkey intending to take the pH1N1 vaccine [20], [24]. Second, all the studies are cross-sectional rather than longitudinal; none assessed subsequent actual vaccination status. Thus, although associations have been identified, there is no way to infer causality. Third, most of the studies are atheoretical. Although some of the studies developed their study questions based on theoretical framework such as HBM [17], [23], [24], none have conducted model analysis and evaluated the model fit. Therefore, due to these three reasons, there remains a significant concern about how valid such results are and a significant knowledge gap about how the observed pattern of influences could be explained.

### Intention-behaviour relation

A major limitation of previous empirical studies [12], [15]–[23], [25], [26] is failure to accommodate the intention-behaviour gap. Although several behavioral theories such as Protection Motivation Theory (PMT) [34], [35], Theory of Reasoned Action (TRA) [36], [37] and Theory of Planned Behaviour (TPB) [38], [39] propose that intention is the proximal determinant of behaviour, intention does not necessarily translate into actual behaviour. Empirical studies of the intention-behavior relationship showed that intention had a medium effect (a correlation of ∼0.4–0.5) on behavior [40]–[42], but a recent review including 47 experimental studies found that a medium-to-large change in intention induced by manipulated interventions caused only a small-to-medium change in behavior [43], where an effect size of 0.5 is medium and one of 0.2 is small [44]. Sheeran found that about 47% of those intending to take action fail to act [42], consistent with Harris et al's findings [33]. Factors that are prime contenders to moderate/mediate the relationship between intention and behaviour include behavioural control/efficacy, action planning and anticipation of consequences [41]–[43].

#### Perceived behavioural control/self-efficacy

The TPB is an extension of the TRA incorporating the concept of perceived behavioural control (PBC) as an intervening variable predicting both intention and also actual behavioural change directly [38], [39]. The direct effect of PBC on actual behavioural change partly explains why not all intention translates into behaviour. Previous reviews suggested that intention-behaviour relationships could be moderated by perceived behavioral control, with higher levels of perceived behavioural control improving prediction of intention on behaviour [42], [43]. Although some researchers suggested that PBC differs from self-efficacy because self-efficacy emphasized perceived internal control more while PBC also considers external control factors [45], a systemic review on the efficacy of TBP found that PBC and self-efficacy had comparable effects on intention and behaviour [41]. Despite being a dominant theory of behavioural change, because the TPB is limited in predicting behaviour we sought to enhance its predictive power by replacing PBC with self-efficacy and incorporating enhanced social effects to accommodate external control factors.

#### Implementation of intention/planning

Implementation of intention, termed “planning”, is a potentially important factor facilitating translation of intention into behaviour [42], [43], [46], [47]. Planning is specific to situations (e.g., when, where, and how) within which one will perform the behaviour [46]. It activates the situational context for goal attainment and thereby makes the goal become more accessible [46], [47]. A meta-analytic review showed that implementation of intention as planning consistently caused a medium-to-large effect on behavioural change [47].

#### Anticipated regret

Anticipated regret is the expectation of feeling regret or upset if one does or does not conduct certain behaviours. Anticipated regret has been found to be a strong predictor of vaccine uptake against seasonal influenza [31], [32], playing the lottery [48] and exercise [49]. Anticipated regret might also moderate the intention-behavior relationship: the higher anticipated regret for inaction, the better the prediction of intention on behaviour [48], [49].

### Our conceptual model

A robust theoretical framework comprehensively explaining behavior change that elucidates population decision-making for health protective and promoting behaviour has long been sought. As the main contender, the TPB explains ∼34% of variance in health behavioural change related to addictive behaviour, automobile-related behaviours, clinical and screening behaviour, eating behaviour, exercising behaivour, HIV/AIDS-related behaviour and oral hygiene behaivour [40]. The standard version of TPB proposes that attitudes towards the behaviour, subjective norm and PBC predict behavioral intention while intention and PBC predict the actual behavioural change [38], [39]. Additional predictors that significantly improve the model's predictive power are needed [39]. Two previous studies have examined modified versions of TPB to predict vaccination uptake against seasonal influenza [50], [51]. One study used TPB plus two additional factors: influenza vaccination history and anticipated regret, to predict intention to receive vaccine against seasonal influenza among elderly from social clubs [50]: vaccination history and anticipated regret respectively accounted for an additional 10.7% and 13.7% of total variance in influenza vaccination intention [50]. However, again the study was cross-sectional and actual vaccination uptake was not assessed. A second study of healthcare workers [51] adopted an extended version of TPB that included additional elements of anticipated regret, moral norm, descriptive norm and professional norm. The study found that controlling for the original TPB variables, moral norm and anticipated regret were significant determinants of actual receipt of seasonal influenza vaccine [51]. The study provides useful information for future application of the extended version of TPB. However, since the study was conducted among healthcare workers, some of the variables such as moral norm and professional norm which emphasize obligation and professional convictions may not be applicable among the general population.

**Figure 3:**
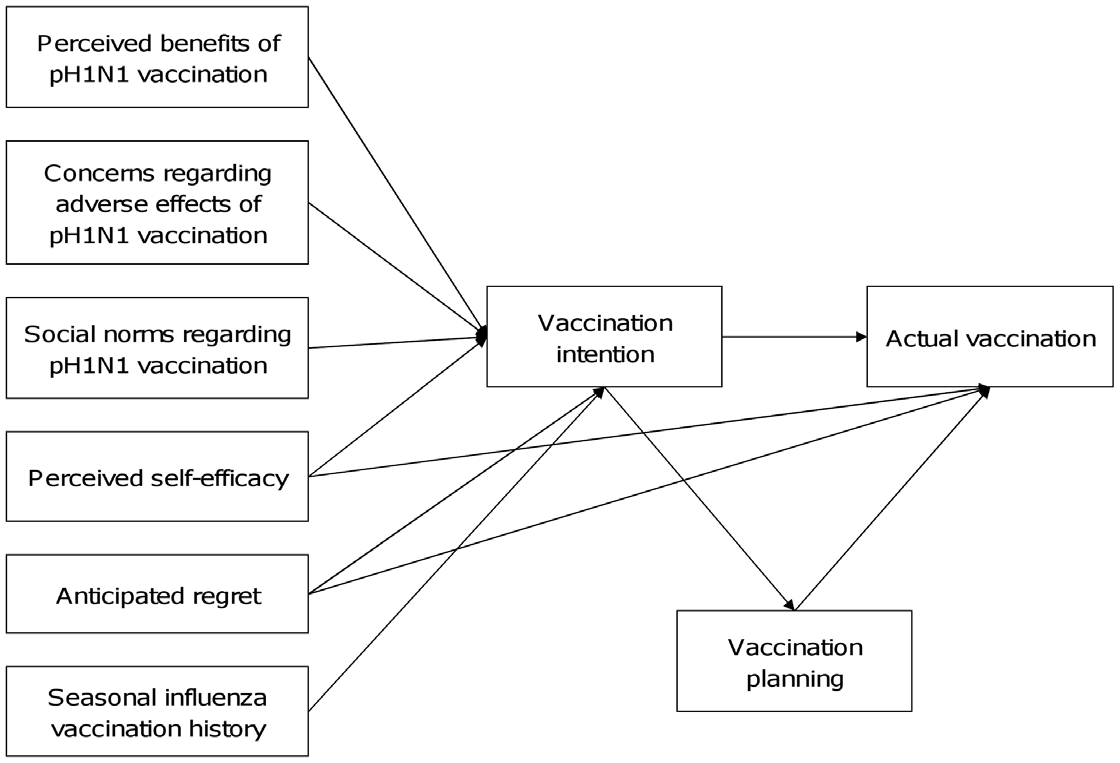
An extended Theory of Planned Behaviour.

Factors influencing pH1N1 vaccine uptake at the later stage of a pandemic might be more cognitively driven unlike behavioral responses during the early stage of a pandemic which might be more affect driven [52]. Therefore, taking into account prior work on seasonal influenza vaccination uptake [50], [51], extending the TPB could provide theoretical utility for understanding public decision on taking pH1N1 vaccination. Starting with TPB and existing literature, we therefore built a conceptual model of public decision-making for pH1N1 vaccination (Figure 3). In addition to the original TPB components, seasonal influenza vaccination history, anticipated regret and vaccination planning were included in the model. The model proposed that attitudes towards vaccination (perceived benefits of pH1N1 vaccination and concerns regarding possible adverse effects of pH1N1 vaccination), perceived social pressures from significant others and other people around regarding pH1N1 vaccination (social norms regarding pH1N1 vaccination), perceived self-efficacy in taking vaccination (perceived self-efficacy), anticipated regret for not taking the pH1N1 vaccination (anticipated regret) and seasonal influenza vaccination history would predict vaccination intention, which in turn predicts vaccination planning and future vaccination uptake; anticipated regret and perceived self-efficacy could also predict vaccination status directly; finally, vaccination planning was proposed to bridge the intention-behavior gap and predict vaccination status directly (Figure 3).

We conducted a longitudinal study of influences on pH1N1 vaccination behaviour in Hong Kong to test this model (Figure 3), and subsequently followed up participants to record their self-reported receipt of pH1N1 vaccine. In this study, we aimed to answer the following research questions: How well does intention predict future uptake of pH1N1 vaccine? Does vaccination planning mediate the relation between intention and future vaccination uptake? And do the original TPB components and the additional components (extended social norms, anticipated regret and seasonal influenza vaccination history) contribute to peoples' decisions on vaccination uptake?

## Methods

### Ethics statement

The study obtained ethics approval from the Institutional Review Board of the University of Hong Kong/Hospital Authority Hong Kong West Cluster. Written informed consent was waived by the IRB because all the data were analyzed anonymously, but verbal consent was obtained from all the subjects before the interview started.

### Sampling

Hong Kong has 99% landline telephone penetration, local calls are free and telephone interviews are common and representative methods of survey data collection [53]. We conducted 13 main cross-sectional telephone surveys of psychological and behavioural responses to the first wave of the 2009 influenza A/H1N1 pandemic in Hong Kong from April through November 2009 (the parent study) [53] in order to monitor these variables. As an extension, the present study re-contacted subjects from some of these surveys and sought to understand public decision-making regarding pH1N1 vaccine uptake for mitigating the potential second wave of the pandemic. Between 12–25 January, 2010 a baseline assessment for the present study was performed, immediately prior the local pH1N1 vaccination campaign extending to the general community (vaccination for high risk groups started from December 21, 2009), and we again contacted participants for follow-up two months later, between 15–30 March 2010.

#### Sample size determination

We estimated that a sample of at least 500 was required to achieve 80% power at an α = 0.05 to reject a model of the specified complexity (Figure 3) if the model fit index Root Mean Square Error of Approximation (RMSEA) exceeded 0.08 [54], [55]. To allow for a response rate ∼60% in the follow-up and the baseline surveys, we need to target at least 1,389 subjects in the baseline survey.

#### Subject selection and inclusion criteria

**Figure 4:**
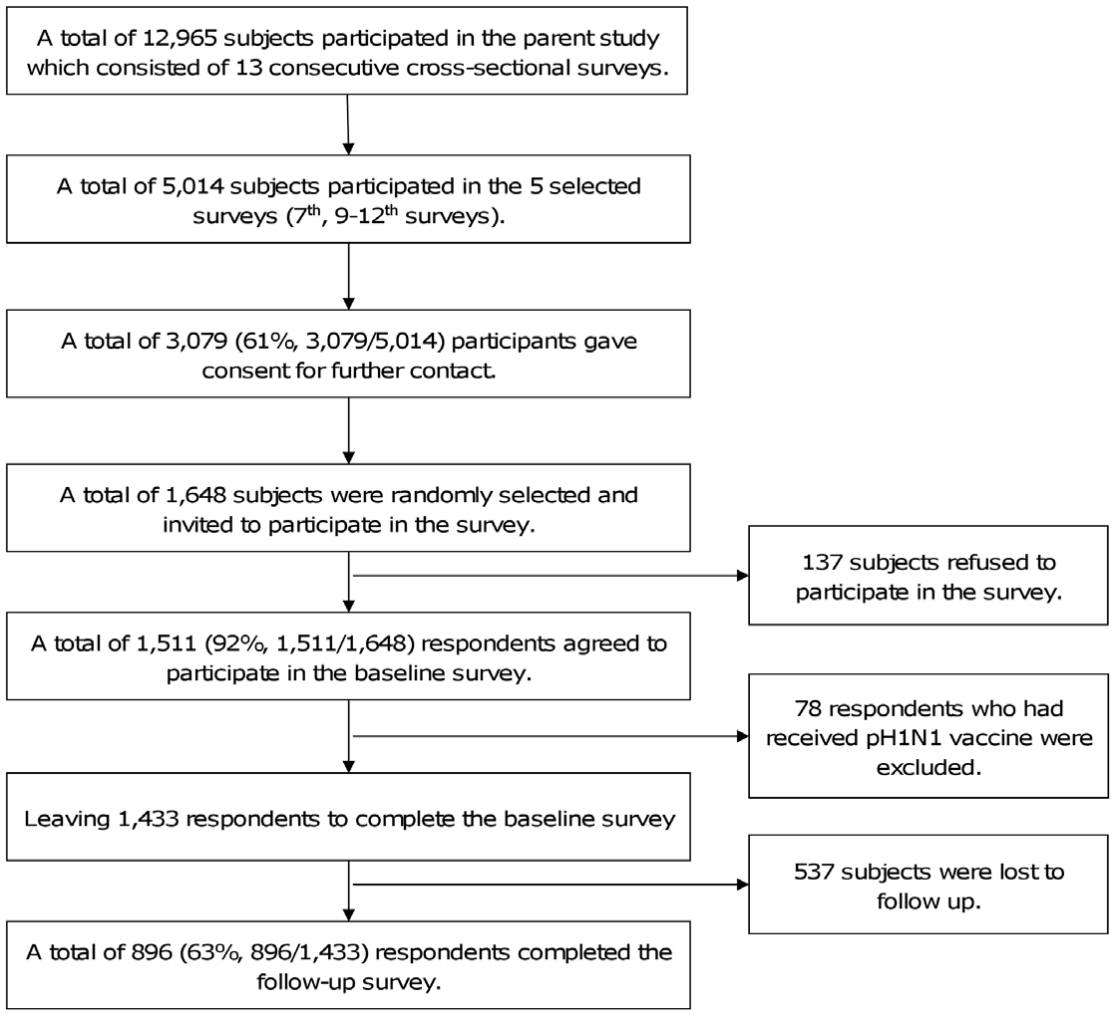
Flow chart of sampling.

A flow chart showing subject selection is provided in Figure 4. A total of 12,965 subjects participated in the parent study [53]. All these subjects were Cantonese-speaking adults (aged≥18) selected within households using a Kish Grid methodology, who were capable of and willing to answer a telephone interview. Additional details about inclusion criteria are available elsewhere [53]. Respondents in the 7^th^, 9–12^th^ surveys of the 13 surveys comprising the parent study who, in the parent study agreed to be re-contacted and who had not received pH1N1 vaccine were invited to complete the baseline assessment for the present study. These five surveys (the 7^th^, 9–12^th^ surveys) were selected because participants in these surveys had not had any follow-up contact either in the parent study or otherwise. This minimizes interview fatigue thereby improving response rates. These surveys were all of a comparable sample size, between 1,000–1,007 [53]. The five selected surveys were conducted between 21 July and October 23, 2009, and generated a representative [53] pool of 5,014 respondents of whom 61.4% (3,079/5,014) gave consent for further contact. From a list of the 3,079 subjects who agreed to be re-contacted, 1,648 calls were randomly selected and successfully made by a university telephone polling organization. Unanswered calls were tried at least four times at different hours and weekdays before being replaced by new numbers. Finally, a total of 1,511 (92%, 1,511/1,648) respondents agreed to participate in the baseline survey. Of these 78 (5%, 78/1,511) reported already having received pH1N1 vaccination and were therefore excluded as ineligible, leaving 1,433 respondents who completed baseline interviews.

### Data collection

The interview questionnaire for the baseline survey was derived from literature review, our previous cross-sectional surveys [53] and the theoretical framework constructed for this study (Figure 3). Specialists in health psychology, statistics, infectious disease and public health jointly determined the measures comprising the final questionnaire, guided by the need to maintain low assessment load and parsimony to ensure good response rates. The finalized questionnaire consisted of five sections: Section 1 addressed respondents' self-rated health and their experience of influenza-like illness in the past six months; Section 2 addressed risk perceptions regarding pH1N1; Section 3 addressed perceived trust in information related to pH1N1 and pH1N1 vaccination from different information sources; Section 4 addressed attitudes, beliefs and social norms regarding pH1N1 vaccine/vaccination, vaccination intention and planning; Section 5 addressed key respondent demographics. Overall, the baseline assessment consisted of 44 questions, which took less than 15 minutes to complete. Other demographic data were obtained from the parent study [53]. Prior to baseline assessment for the present study, subjects were reminded of their prior participation and that they had agreed to participate in a further study. The study was introduced as a survey of attitudes towards swine flu vaccination. We sought their willingness to participate. Those agreeing were asked about their vaccination status. Subjects who reported that they had already received pH1N1 vaccination were excluded. The remaining interview was performed. A follow-up survey was conducted 2 months later wherein respondents were reminded of the earlier survey and asked about their vaccination status and reasons for having had or not having vaccination. All the data were collected through telephone interview in both Baseline and Follow-up surveys.

### Measures

The measures comprising the study instruments were used to build the conceptual model (Figure 3) and are described below and in Table 1.

**Table 1:** Items, response scales and internal consistency for assessing measures of model.

Measures	Items	Response scales	α^a^
Perceived benefits of pH1N1 vaccination	I believed that the HSI^b^ vaccine can protect me against HSI.	1–5 agreement	0.71
	I believe that it will help to protect my family or friends against HSI if I take the HSI vaccination	1–5 agreement	
	I believe that the HSI vaccination can reduce my risk of contracting HSI.	1–5 agreement	
Concerns regarding adverse effects of pH1N1 vaccination	I fear that the HSI vaccination will cause some unpleasant side effects.	1–5 agreement	0.64
	I worry that the vaccine may cause more harm than the flu	1–5 agreement	
Social norms regarding pH1N1 vaccination	Other people going to take HSI vaccination will encourage me to go.	1–5 agreement	0.53
	My family and friends think that it is important for me to take vaccination against HSI	1–5 agreement	
Anticipated regret	If you decide not to take the HSI vaccination this winter, how likely will you regret your decision?	1–7 likelihood	0.68
	If you decide not to take the HSI vaccination this winter, and later you were infected with HSI and infect other household members, then how likely do you think it is that you will regret your decision?	1–7 likelihood	
Perceived self-efficacy	I am confident that I can go independently to get HSI vaccination.	1–5 agreement	-
Seasonal influenza vaccination history	Have you received seasonal influenza vaccination?	Yes/No	-
Vaccination intention	How likely is it that you are going to have the HSI vaccination this winter?	1–7 likelihood	-
Vaccination planning	I have planned when and where to get my HSI vaccination this winter.	1–5 agreement	0.59
	When vaccines are available I intend to discuss with my doctor if s/he thinks it is good for me to have the vaccination	1–5 agreement	
	I have discussed with my family about my plan for HSI vaccination	1–5 agreement	

a Chronbach's α indicates the internal consistency.

b HSI represent Human Swine influenza, the local colloquialism for pH1N1.

#### Perceived benefits of pH1N1 vaccination, and, Concerns regarding adverse effects of pH1N1 vaccination

These two constructs assessed attitudes towards pH1N1 vaccination. Perceived benefits of pH1N1 vaccination was assessed by measuring agreement on five-point ordinal scales (from 1 “strongly disagree” to 5 “strongly agree”) with three statements (Table 1). A Cronbach's alpha (α) of 0.71 indicated an acceptable internal consistency for this scale and these two items were treated as the indicators of a latent scale (Perceived benefits of pH1N1 vaccination). Concerns regarding adverse effects of pH1N1 vaccination were assessed by measuring agreement, using five-point scales, with two statements. The Cronbach's α for these two items was 0.64, considered acceptable by some researchers [56], though clearly less than desirable. We therefore treated the items as reflecting a latent variable (Concerns regarding adverse effects of pH1N1 vaccination).

#### Social norms regarding pH1N1 vaccination

While TBP considers the influence of solely coercive social pressure from significant others to perform a behaviour, previous studies suggest that it is also important to consider the generalized tendency to adopt behaviours demonstrated by others encountered in daily life for imitative reasons [48], [57]. We use the term Social norms rather than subjective norm to represent these broader coercive and imitative social influences. Social norms were assessed by agreement on a 5-point scale with two statements. The internal consistency for these two items was weaker, with α = 0.53, which suggests each item appropriately measures different social influences. We initially incorporated these items separately in the structural equation model but except for the path weights dividing almost equally between the two items, no difference was otherwise seen. We therefore retained them as indicators of a combined latent construct in the model for purposes of model parsimony [58].

#### Anticipated regret

Anticipated regret was assessed with two statements asking about respondents' likelihood of feeling regret. Responses of these two items were on a 7-point categorical scale (from 1 “definitely not” to 7 “certain”). The internal consistency α for these two items was 0.68. The two items were used to indicate the latent variable “anticipated regret” in the modeling analysis.

#### Perceived self-efficacy

One item was used to measure self-efficacy, asking about respondents' agreement on a 5-point scale with the statement “I am confident that I can go independently to get human swine flu vaccination”. A standard scale of self-efficacy was not adopted to minimize assessment load. However, a single item for self-efficacy has been shown elsewhere to have validity in predicting behavioural change [59], [60].

#### Seasonal influenza vaccination history

Respondents were asked whether they had received any seasonal influenza vaccination in the past three years (Yes/no/don't know).

#### Vaccination intention

Respondents were asked how likely it was that they would get vaccinated against pH1N1 during the winter flu season, using a 7-point Likert scale (from 1 “definitely not” to 7 “certain”).

#### Vaccination planning

We measured vaccination planning by assessing respondents' agreement on a 5-point scale with three statement items, such as “I have planned when and where to get my human swine flu vaccination this winter”. The internal consistency α for these three items was 0.59, though less than the most common acceptable level of above 0.7, remaining at the minimal acceptable level (α ranged between 0.5–0.6) of reliability for preliminary research [56]. These items were also treated as indicators of a latent variable for modeling purposes.

#### Reported vaccination uptake

In the follow-up survey, respondents were asked to confirm if they had received pH1N1 vaccine within the past three months. Respondents were also asked to indicate their major reasons for having or not having taken the pH1N1 vaccination using open-ended questions. Multiple reasons could be given by each respondent.

### Statistical analysis

We first compared demographic differences between follow-up and lost-to-follow-up respondents with Pearson chi-square test while demographic differences of the respondents who completed both the baseline and follow-up survey and the general population [61] were assessed using Cohen's effect sizes [44]. Proportions were calculated to describe patterns of vaccination intention, reported vaccination uptake, and major reasons for taking or not taking pH1N1 vaccination. Structural equation modeling was then applied to examine the determinants of pH1N1 vaccination, vaccination intention and vaccination planning based on the extension of TBP. Mplus 6.0 for Windows (Muthén & Muthén, 1998–2010) was employed because the model comprised dichotomous (vaccination status) and ordinal (vaccination intention) outcome variables. Before testing the full structural model, zero-order correlations between the measures of related constructs were calculated. Confirmatory factor analysis was performed to assess the adequacy of the measurement model including perceived benefits of pH1N1 vaccination, concerns regarding adverse effects of pH1N1 vaccination, social norms regarding pH1N1 vaccination, anticipated regret and vaccination planning. To test the full structural model, all variables were entered into the model simultaneously. Mean and variance adjusted weighted least squares estimation was applied to evaluate the standardized parameters (beta, β). Since chi-square test is very sensitive to sample size and non-normally distributed data, several other model fit indices were evaluated including the Comparative Fit index (CFI), the Tucker Lewis index (TLI), and RMSEA. A CFI>0.90 and TLI>0.90 indicates a good fit. RMSEA less than 0.05 and one ranging between 0.05–0.08 respectively indicate a good and acceptable model fit [55]. Misfitting models were re-specified guided by theoretical soundness and modification indices [55]. Missing proportions ranged from 0.1% for seasonal flu vaccination history to 5.5% for the item “I have planned when and where to get my pH1N1 vaccination this winter”. There was no missing data for reported vaccination uptake. Missing data were handled with multiple imputation [62].

## Results

### Participants

Of the 1433 respondents who completed the baseline assessment, 896/1433 (63%) respondents agreed to participate and completed the March follow-up survey (Figure 4). Demographic characteristics of respondents in the baseline and follow-up surveys are shown in Table 2. Compared to respondents completing both baseline and follow-up surveys, respondents lost to follow-up were younger (χ^2^ = 14.24, p = 0.001) and more likely to be single (χ^2^ = 20.26, p<0.001). Overall, the low Cohen effect sizes (<0.3) showed that the demographics of respondents who completed both the baseline and follow-up surveys were comparable to those of the general population of Hong Kong [61].

**Table 2:** Comparison of the demographics of respondents and non-respondents in the follow-up survey.

Demographics	Baseline (n = 1433)	Follow-up (n = 896)	Lost to follow-up (n = 537)	χ^2^ a	Effect size^b^
Gender (Female)	63%	63%	63%	0.038	0.22
Age group					
18–34	26%	23%	31%	14.24^c^	0.22
35–54	45%	45%	44%		
≥55	29%	32%	25%		
Education level					
Primary or below	15%	15%	14%	3.40	0.27
Secondary	51%	52%	49%		
Tertiary or above	34%	32%	37%		
Marital status					
Single	31%	27%	38%	20.26^c^	0.11
Married	64%	68%	56%		
Divorced/separated/widowed	5%	5%	6%		
Birth place (Born in Hong Kong)	71%	71%	72%	0.37	0.22
Household income					
≤10,000	21%	21%	20%	6.45	-
10,000–20,000	27%	29%	23%		
20,000–30,000	21%	21%	22%		
≥30,000	31%	29%	34%		

a Demographics differences between follow-up respondents and those who were lost to follow up.

b Effect sizes 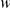 are calculated via the formula 
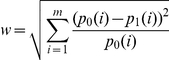
, where 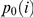 and 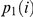 are the observed proportions in the i'th category from the 2006 Hong Kong by-census data and the follow-up data, respectively.

c p<0.01.

### Vaccination intention at baseline and vaccination status at follow-up

Of the 1,433 respondents who completed the baseline survey, 36% (510/1,433) reported that they would “definitely not” take pH1N1 vaccination during the winter flu season; 36% (521/1,433) reported being “very unlikely/unlikely” to take it; 19% (278/1,433) reported their pH1N1 vaccination likelihood as “evens” (50∶50/equal likelihood); and only 8% (119/1,433) reported vaccination likelihood as “likely/very likely/certain”. Within the subset of 896/1,433 respondents who completed both baseline and follow-up surveys, 7% (67/896) had reported at baseline that they would be “likely/very likely/certain” to receive pH1N1 vaccination. However, in the follow-up survey, only 7/896 (0.8%) respondents reported having received pH1N1 vaccination in the intervening period, 4 of whom had reported being “likely/very likely/certain” to receive pH1N1 vaccination at baseline. Reporting higher intention to receive pH1N1 vaccination in the baseline was associated with greater likelihood to vaccinate by follow-up (Fisher's exact test, χ^2^ = 24.24, p<0.001).

### Major reasons for receiving pH1N1 vaccine

The 7 respondents who reported taking pH1N1 vaccination gave the major reasons for deciding on vaccination as follows: Three choose vaccination because of the ‘*high risk of swine influenza*’ characterized by statements like “swine flu is serious”, “I am worried that swine flu will become more serious”, and “I feel vulnerable to swine flu”; two reported that their decision was due to ‘*doctors' advice*’ and two reported ‘*belief of the vaccine efficacy*’. Other reasons provided by one respondent only were ‘*belief in the vaccine's safety*’, ‘*government recommendation*’, ‘*convenient availability*’, and ‘*protection of patients*’.

### Major reasons for not receiving pH1N1 vaccine

**Figure 5:**
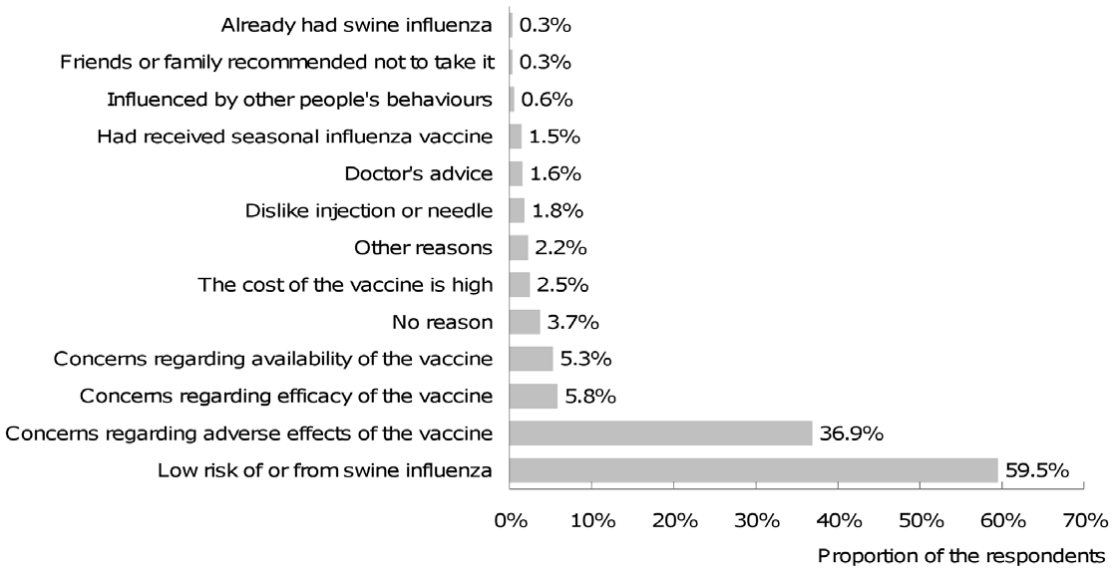
Major reasons for rejecting pH1N1 vaccination among respondents who reported not receiving pH1N1 vaccination.

Reasons for not having vaccination given by the 889 respondents who did not receive pH1N1 vaccination (Figure 5) were, most frequently ‘*low risk of or from swine influenza*’ (529/889, 60%) and ‘*concerns regarding adverse effects of the vaccine*’ (328/889, 37%). Around 11% (100/889) of the respondents reported both ‘*low risk of/from swine influenza*’ and ‘*concerns regarding adverse effects of the vaccine*’.

### Structural equation model for receipt of pH1N1 vaccine

Table 3 presents the means, standard deviations, factor loadings and correlations between the construct measures in the full structural equation model. All the standardized factor loadings exceeded 0.49 and were statistically significant. The model fit indices for the measurement model indicated a good fit with CFI = 0.95, TLI = 0.93, and RMSEA = 0.05.

**Table 3:** Correlations, means, standard deviations, and standardized factor loadings for the measurement model.

Measures	1	2	3	4	5	6	7	8	9	10	11	12	13	14	15	16
1. Ben 1	1															
2. Ben 2	.447^b^	1														
3. Ben 3	.562^b^	.453^b^	1													
4. Con 1	−.176^b^	−.113^b^	−.182^b^	1												
5. Con 2	−.238^b^	−.140^b^	−.219^b^	.476^b^	1											
6. Nor 1	.273^b^	.347^b^	.276^b^	−.085^a^	−.103^b^	1										
7. Nor 2	.285^b^	.325^b^	.262^b^	−.169^b^	−.147^b^	.363^b^	1									
8. Reg 1	.236^b^	.247^b^	.194^b^	−.021	.001	.328^b^	.208^b^	1								
9. Reg 2	.270^b^	.282^b^	.216^b^	.012	−.028	.310^b^	.202^b^	.518^b^	1							
10. Eff	.175^b^	.135^b^	.176^b^	−.142^b^	−.129^b^	.119^b^	.111^b^	.061	.059	1						
11. PFl	.036	−.009	.035	.047	.064	.049	.042	.099^b^	.063	−.033	1					
12. Int	.280^b^	.350^b^	.220^b^	−.194^b^	−.166^b^	.413^b^	.347^b^	.481^b^	.395^b^	.191^b^	.152^b^	1				
13. Pla 1	.225^b^	.252^b^	.182^b^	−.189^b^	−.114^b^	.365^b^	.407^b^	.207^b^	.180^b^	.138^b^	.132^b^	.451^b^	1			
14. Pla 2	.243^b^	.336^b^	.172^b^	−.040	−.038	.282^b^	.236^b^	.203^b^	.188^b^	.083^a^	.126^b^	.334^b^	.343^b^	1		
15. Pla 3	.146^b^	.178^b^	.120^b^	−.009	−.016	.264^b^	.235^b^	.141^b^	.075^a^	.102^b^	.149^b^	.237^b^	.395^b^	.226^b^	1	
16. Vac	0.02	0.02	0.03	−0.09^a^	−0.03	0.04	0.08^a^	0.11b	0.03	0.02	0.09^b^	0.13^b^	0.14^b^	0.05	0.08^a^	
Means^c^	3.26	3.25	3.41	3.63	3.22	2.58	2.53	2.26	3.48	3.59	19%	2.57	2.14	3.13	2.38	0.8%
SD	0.90	0.89	0.87	0.94	1.04	0.94	0.88	1.42	1.88	0.95	-	1.42	0.81	1.07	0.94	-
Loading	1.00	0.64	0.72	1.00	0.74	1.00	0.58	1.00	0.72	-	-	-	1.00	0.49	0.50	-

Ben = perceived benefits of PH1N1 vaccination, Con = concerns regarding adverse effects of PH1N1 vaccination, Nor = social norms regarding PH1N1 vaccination, Reg = anticipated regret, Eff = perceived self-efficacy, Pfl = past influenza vaccination behaviour, Int = PH1N1 vaccination intention, Pla = PH1N1 vaccination planning, Vac = Vaccination status.

a p<0.05,

b p<0.01,

c Means of dichotomous variables are replaced by proportions of “ones” observed.

**Figure 6:**
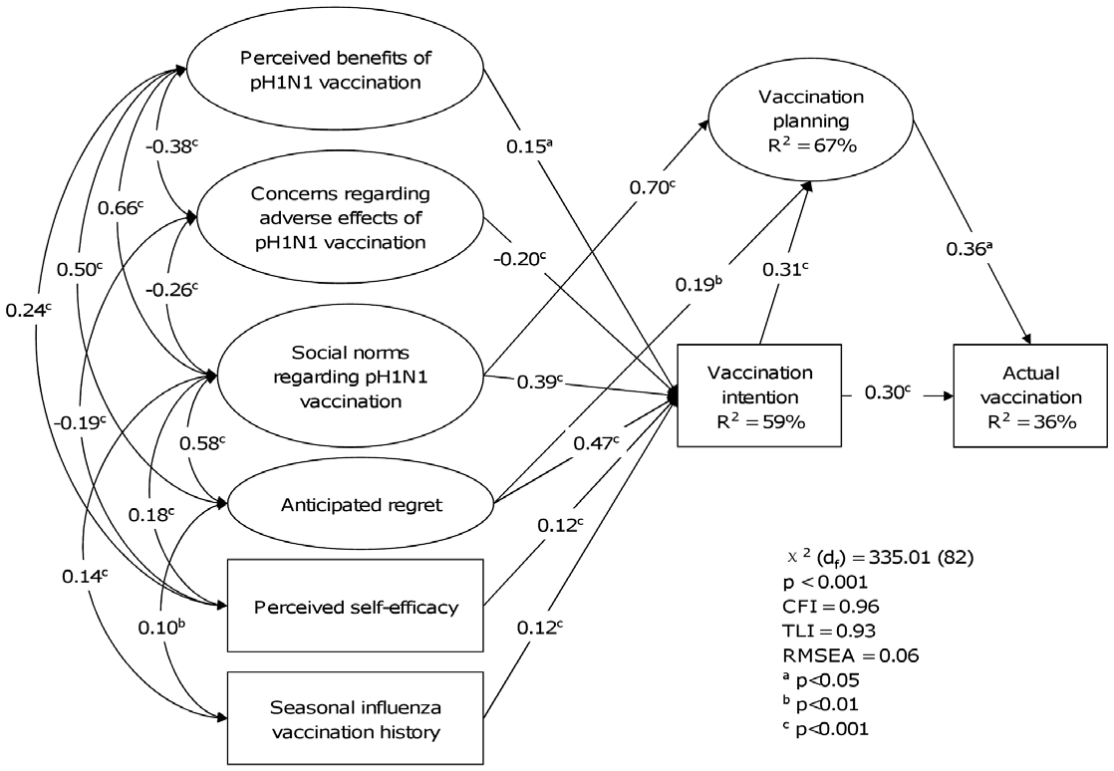
Structural Equation Model of pH1N1 vaccination uptake. Numbers represent the standardized parameters (β). R^2^ represents the explained variance of the dependent variables by the predictors (Sample size N = 896). Ovals represent latent variables, rectangles represent observed variables.

For the final full structural model (Figure 6), two additional paths were added and estimated based on the modification indices including a path from social norms to vaccination planning and path from anticipated regret to vaccination planning while the path from perceived self-efficacy to vaccination and the path from anticipated regret to vaccination were removed, coefficients for these two paths being non-significant and too small to be meaningful. The final model indicated a good fit with CFI = 0.96, TLI = 0.93 and RMSEA = 0.06 (Figure 6).

The model showed that respondents perceiving greater pH1N1vaccination benefits (β = 0.15), less concerns regarding vaccine adverse effects (β = −0.20), greater sensitivity to social norms β = 0.39), higher anticipated regret if not vaccinated (β = 0.47), higher perceived self-efficacy in taking pH1N1 vaccination (β = 0.12) and receiving seasonal influenza vaccination in the past three years (β = 0.12) reported greater intention to take pH1N1 vaccination, and accounted for 59% of variance in vaccination intention scores. Greater adherence to social norms (β = 0.70), higher vaccination intention (β = 0.31) and higher anticipated regret (β = 0.19) were associated with more vaccination planning, together accounting for 67% of variance in vaccination planning. Both vaccination intention (β = 0.30) and vaccination planning (β = 0.36) significantly predicted actual pH1N1 vaccination, accounting for 36% of variance in pH1N1 vaccination (Figure 6).

## Discussion

The World Health Organization recommended a stepwise use of pH1N1 vaccines for protecting people against the pH1N1 influenza pandemic in July 2009 [63]. However, a vaccination program's efficacy largely depends on the public's compliance. Our study found that only 5% of 1,511 subjects reported having received pH1N1vaccination and of 1,433 subjects remaining unvaccinated, only 8% reported intending (being likely/very likely/certain) to take the pH1N1 vaccine. Two months later in the follow-up survey, an even smaller proportion, 0.8% of the respondents who completed both the baseline and follow-up survey reported having been vaccinated against pH1N1. Perceived low risk of pH1N1 and concerns regarding vaccine-related adverse effects were the two most frequently cited reasons for refusing the vaccination. The extended TPB model suggests that both the original TPB components and the additional components contribute to people's decisions on vaccination uptake but that social norms and anticipated regret for not taking vaccination were the strongest determinants of vaccination intention and vaccination planning. Finally vaccination planning partially-mediated the relation between intention and reported vaccination uptake.

Compared to previous studies, vaccination intention was much lower in our study than that found in surveys conducted prior to the influenza pandemic [23] or before the vaccine was available [11], but was comparable to the findings of surveys conducted in France [20] and Turkey [24] after pH1N1 vaccination programmes were launched there. An earlier Hong Kong study that relied on expressed intent to predict vaccination uptake [11] failed to accurately predict the subsequent meager population uptake of pH1N1 vaccination by, at best, an order of magnitude [64], suggesting that intention alone is insufficient for predicting future vaccination uptake, consistent with empirical findings in other areas [33], [42].

Despite predictions that intended pH1N1 vaccination uptake would decline if there was insufficient data on novel vaccine safety and efficacy [11], safety issues were not the predominant barrier to vaccination in the present study. While 37% of our study respondents who remained unvaccinated cited vaccine safety concerns, despite good evidence that the vaccine is effective with a risk profile similar to that of seasonal influenza vaccine [65], almost twice as many, 60%, cited ‘low risk of/from swine influenza’ as their reason for not getting vaccinated, suggesting that these respondents felt no advantage would be gained by vaccination. Around 11% of respondents, cited both ‘low risk of/from swine influenza’ and ‘concerns regarding adverse effects of vaccine’ as the reasons for not getting vaccinated, seemingly adopting a risk-benefit approach to vaccination decision-making. However, in the setting of low influenza risk, with the reports of vaccine related adverse events in the media after the vaccine was available for the priority groups (Figure 2), people may shift their perceived risks away from influenza and towards vaccination, suggestive of availability bias (risk distortion by easily recalled events) [66]. We believe that perceived vaccine risk would become progressively less of a barrier to vaccination as perceived influenza risk increases, and vice versa.

Moreover, despite recent reports that Hong Kong residents would be sensitive to vaccination pricing when considering whether to vaccinate [11], [12], only 2.5% of our respondents cited high vaccine cost as the reason for rejecting vaccination.

Major reasons for taking pH1N1 vaccination corresponded to reasons for not taking it, with perception of pH1N1 risk most frequently cited. However, the few respondents receiving pH1N1 vaccination prohibited meaningful comparison.

The extended version of TPB model fits well to the survey data. The model showed that an expanded social norms and anticipated regret accounted for most of the variance in vaccination intention, rather than the more core elements of TPB. In turn, social norms independently accounted for more than twice the variance in vaccination planning than did intention, and vaccination planning accounted for more variance in vaccination uptake than did intention. Thus it seems that social norms comprise the major influences on vaccination uptake through modifying vaccination intention and planning. A meta-analytic review of TPB efficacy concluded that the TPB variable subjective norm (perceived coercive social pressure from significant others) weakly predicted intention compared to other TPB components, mainly due to poor measurement [41]. “Descriptive norm” (perception of what other people do, imitation or conformity behaviour) is reportedly a more important predictor for intention [48], [57]. Here we combined measures of subjective and descriptive norms, treated as a latent variable (social norms), because they were found to have much the same predictive direction and weight. Multiple item measures of norms should have better predictive power than single item measures [41].

This model importantly informs public health approaches to population behaviour during respiratory epidemics. First, information uncertainty or untrustworthiness, for example regarding vaccine safety, is likely to prompt people look to others for their cues to action: the social environment, namely what other people believe and do powerfully influences decisions to action [45], [67]. People often tend to imitate others, so establishing a “vaccination trend” may help uptake. For example, it could be effective to encourage those who remain unvaccinated with feedback from vaccinated peers and by providing an updated total of numbers vaccinated. What the general public think and do may prove to be as influential as information from scientists or health professional [68], [69]. Second, encouraging uptake of a new vaccine will be problematic if the associated threat element is low, irrespective of vaccine pricing, particularly for novel and untested vaccines. Vaccine safety and efficacy data should be provided wherever possible at all levels including through health-care providers, media and the general public. To effectively communicate the risk and benefit of a novel vaccine, it is important to establish an effective surveillance system to monitor vaccination progammes and rapidly respond to any reported adverse events [70]. The media have an important influence and both reactionary and opinionated news items should be recognized as potentially detrimental to vaccination uptake. In particular, the need to develop stories that generate revenue increasingly overrides balanced reporting in contemporary media. Hence risk amplification remains a problem. Public health agencies need to improve their liaison with influential media outlets to minimize this, where possible. Third, omission bias, a phenomenon where people view vaccination as more risky than remaining unvaccinated, could be a barrier for vaccination [71]. Omission bias arises when there is anticipation of greater regret about adverse effects of vaccination, if taken, than the regret about being infected with influenza if vaccination is rejected [72]. Therefore, social marketing emphasizing the far greater likelihood of regret for consequences due to refusing vaccination than the regret over an improbably low adverse event due to taking vaccination may help to reduce this bias. For example, previous studies found that simply asking two questions about feeling regret for inaction could increase respondents' intention to play a lottery or do exercise [48], [49]. Finally, vaccination planning is a key intervening variable between vaccination intention and actual vaccination. This is to be expected given that it is more proximal to actual behaviour than intention is. In those who may be undecided, interventions facilitating planning may prompt action. This could include suggesting where, when and how to get vaccination, improving and publicizing accessibility of vaccination centres and opening times. Even so, intention and planning explained only 36% of the variance in the reported vaccination behaviour, suggesting that other factors, such as intention stability [42], influencing vaccination behaviour await identification.

Study limitations include baseline attitudes/beliefs, vaccination intention and planning being measured at the same time point, prohibiting exploration of causality in observed associations. Some study measures were constrained due to length of telephone interviewing, and while sub-optimal were necessary methodological compromises. Although most researchers recommended Cronbach's α of 0.7 as the minimal acceptable for internal consistency of multi-item scales, others accept 0.6 or 0.5–0.6 for preliminary research as the cut-off point [56]. Other than dimensionality concerns, lower α can reflect too few items comprising the putative scale [73]. This is more likely for complex variables, such as social norms which have a broad spectrum of elements. Though less than perfect, measurement errors can be reduced by incorporating the items as a latent variable in SEM [55], an approach we adopted. Additionally, collinearity between exogenous indicators, such as social norms and perceived benefit can be potentially problematic, perhaps lowering the accuracy of SEM estimation. However, since high associations between measures of the constructs were not observed (Table 3) then collinearity-related error is probably small [74]. Despite being randomly selected for the parent study, subjects of this study were not randomly selected from the general population, although demographics suggest the current sample is comparable to the Hong Kong general population [61] (Table 2). Moreover, subject recruitment was based on voluntariness and all data were self-reported. All could cause social desirability and selection bias, so caution is needed before extrapolation to the general population. Also refusal at follow-up could have influenced patterns of responses. Our study examined public decision-making regarding a novel influenza pandemic vaccine. Our findings may not apply to vaccination against seasonal influenza due to numerous differences in beliefs towards the vaccination. For example, although perceived low risk remains the major reasons for refusing vaccination against seasonal influenza as in our study, vaccine safety is seldom cited as a barrier [28], [29] whereas we found that about one third of respondents had vaccine safety concerns. Cultural differences in influenza and vaccination-related beliefs are possible [75], but these differences may gradually diminish with the increasing identical news information available through the three dominant news agencies and common public health strategies being increasingly universal. Related stories, such as use of preservatives and adjuvants in vaccine manufacture may enhance knowledge and reduce trust in product safety [76]. The role of media remains much under-researched in this regard. Finally, data was insufficient to reliably report the reasons for pH1N1 vaccination uptake among the population.

Nonetheless, compared with other cross-sectional studies [12], [15]–[27], the longitudinal design of this study strengthens understanding of influences on population decision-making for pandemic influenza vaccination uptake and represents a step forward in this area of research. This study is novel in linking theoretically derived, vaccination-related cognitions to subsequent influenza vaccination behaviour, and exemplifies that within the Hong Kong Chinese culture, social norms and action planning are far more influential than intention in predicting vaccination behaviour.
